# Insights into the Cellular Function of YhdE, a Nucleotide Pyrophosphatase from *Escherichia coli*

**DOI:** 10.1371/journal.pone.0117823

**Published:** 2015-02-06

**Authors:** Jin Jin, Ruijuan Wu, Jia Zhu, Shaoyuan Yang, Zhen Lei, Nan Wang, Vinay K. Singh, Jimin Zheng, Zongchao Jia

**Affiliations:** 1 College of Chemistry, Beijing Normal University, Beijing, China; 2 Department of Biomedical and Molecular Sciences, Queen’s University, Kingston, Ontario, Canada

## Abstract

YhdE, a Maf-like protein in *Escherichia coli*, exhibits nucleotide pyrophosphatase (PPase) activity, yet its cellular function remains unknown. Here, we characterized the PPase activity of YhdE on dTTP, UTP and TTP and determined two crystal structures of YhdE, revealing ‘closed’ and ‘open’ conformations of an adaptive active site. Our functional studies demonstrated that YhdE retards cell growth by prolonging the lag and log phases, particularly under stress conditions. Morphology studies showed that *yhdE*-knockout cells transformed the normal rod shape of wild-type cells to a more spherical form, and the cell wall appeared to become more flexible. In contrast, YhdE overexpression resulted in filamentous cells. This study reveals the previously unknown involvement of YhdE in cell growth inhibition under stress conditions, cell-division arrest and cell-shape maintenance, highlighting YhdE’s important role in *E*. *coli* cell-cycle checkpoints.

## Introduction

Maf (multicopy associated filamentation) proteins are part of a large family of conserved proteins found in bacteria, archaea and eukaryotes. Although this family is implicated in the regulation of cell division, their exact cellular function remains unknown. Previous genetic experiments showed that introduction of the *maf* gene on a multi-copy plasmid into *Bacillus subtilis* resulted in extensive filamentation and inhibition of cell division [1], but the biochemical basis for the septation inhibition remains elusive. Recent work on a Maf protein from *B*. *subtilis* indicated that the inhibition of cell division was associated with DNA transformation and repair [2]. The three-dimensional X-ray crystallographic structures of the *B*. *subtilis* Maf protein have been determined in both its apo form and dUTP-bound form [3]. The structural similarity to the *Methanococcus jannaschii* Mj0226 dNTP pyrophosphatase (PPase) [4] and the *Escherichia coli* YjjX ITPase/XTPase [5], both of which are nucleotide hydrolases, provided evidence that Maf is a nucleotide- or nucleic acid-binding protein. A recent study showed that Maf proteins act as dTTPases/UTPases that may be involved in the regulation of DNA/RNA synthesis [6]. However, no direct evidence demonstrating the connection between Maf dTTPase/UTPase and DNA/RNA synthesis or DNA transformation has been reported, and the physiological roles of Maf proteins are still not clear.

A Maf-like protein in *E*. *coli* known as YhdE shares 46% sequence identity with Maf in *B*. *subtilis*. Within the chromosome, the *maf* gene is followed by *mreBCD* and *minCD* [1], while the *yhdE* gene is similarly located with respect to *mreBCD* but is far from *minCD* [7]. In *E*. *coli*, both *mreBCD* and *yhdE* belong to the *mre* operon. It was postulated that MreBCD may act as a scaffold to establish the helical organization of murein-synthesizing proteins in rod-shaped cells [8]. MreB is an actin homolog that acts as a skeleton protein and is located beneath the cytoplasmic membrane in a helical array [9, 10]. The MreB-associated cytoskeleton has been implicated in several cellular processes, including maintenance of cell shape, chromosomal segregation and establishment of cell polarity [11]. MreC and MreD are involved in cell wall synthesis in the cylindrical part of the cell, leading to cell elongation [8]. When bacterial cells are depleted of MreB, MreC or MreD, either individually or as a complex, the cells display a spherical phenotype [12]. Another gene in the *mre* operon downstream of the *yhdE* gene is *rng* (previously called *cafA* or *orfF*). RNase G, which is encoded by the *rng* gene, is a nonessential ribonuclease specific for adenine- and uracil-rich regions [13, 14]. RNase G is homologous to the amino-terminal part of RNase E [15], a protein involved in the regulation of the FtsZ/FtsA ratio [16]. FtsZ and FtsA are proteins involved in septum formation and cell division [17, 18]. Overexpression of RNase G in *E*. *coli* results in the production of cytoplasmic axial filaments that cause the formation of chained cells and minicells, suggesting that RNase G is involved in chromosome segregation and cell division [16].

A comprehensive analysis of previous findings involving the *mre* operon in *E*. *coli* revealed a common phenotypic feature of the *mreB*, *yhdE*, and *rng* genes. Overexpression, but not inactivation, of the products of these genes prevents cell division, resulting in the formation of filamentous cells, indicating that these genes might be involved in cell septum formation or cell division. These observations imply that the genes in the *mre* operon may be cooperatively involved in a common physiological pathway that may play a regulatory role in cell division. However, it is not clear how these genes coordinate with each other both mechanistically and functionally. An understanding of YhdE function would not only shed light on YhdE’s role but also provide novel insights into the regulation of cell division. To date, the physiological roles of YhdE have not been identified with the exception of a recent paper describing the PPase activity of YhdE toward canonical and modified nucleotides [6]. In this study, we confirmed that YhdE is a PPase and provided further evidence that its PPase activity is highly specific, primarily for the deoxyribonucleotide dTTP and secondly for UTP. Structures of YhdE_E33A, an inactive mutant that lacks PPase activity, were determined to elucidate the underlying mechanism and specificity of the PPase activity. To further explore the cellular function of YhdE, we examined the influence of the *yhdE* gene in cell growth under varying conditions and investigated the corresponding changes in cell morphology. Our results reveal the active involvement of YhdE in cell growth inhibition, cell division arrest and cell shape maintenance. Therefore, we propose an important role of YhdE at *E*. *coli* cell-cycle checkpoints.

## Materials and Methods

### Cloning, expression and purification of YhdE

The open reading frame of *yhdE* was amplified by polymerase chain reaction using **GGATCC**ACTTCTCTGTATTTAGCTTCCG as the forward primer and **GAATTC**TCAGCCGTCATGTTTATCCCT as the reverse primer (restriction sites are in bold type). The resulting product was digested with BamHI and EcoRI and ligated into the pFO4 vector. The recombinant YhdE was expressed as an N-terminal hexahistidine-tagged protein in BL21(DE3) *E*. *coli* cells under the T7 promoter.

Site-directed mutagenesis was used to generate pFO4-*yhdE_E33A* using standard procedures with ATTGAGGcGCAGCGTCAGCCGCAGGAGAG as the forward primer and GACGCTGCgCCTCAATGCCCGTAACAATAC as the reverse primer (the mutant site is in lower case). The recombinant YhdE_E33A mutant was expressed in BL21(DE3) *E*. *coli* cells.

Expression and purification of YhdE and the YhdE_E33A mutant were performed following the procedures previously reported for YjjX [5].

### Preliminary investigation of YhdE activity

The standard solution consisted of 6.25 μM YhdE, 5 mM Mg^2+^, 20 mM Bis-Tris buffer pH 6.75 and 100 μM or 500 μM substrate in a 30 μL volume incubated for 10 minutes at 25°C. The reactions were terminated by the addition of 70 μL of a color-developing reagent consisting of six parts 0.42% ammonium molybdate in sulfuric acid with one part 10% (w/v) ascorbic acid. The samples were incubated for 20 minutes at 45°C. Any inorganic phosphate liberated by YhdE was detected by absorbance at 660 nm. The absorbance was determined with a Powerwave XS Microplate spectrophotometer (Bio-Tek Instruments, Inc.).

### Substrate specificity of YhdE

The substrate specificity of YhdE was analyzed by comparing YhdE enzymatic activity in the presence of a variety of concentrations of different nucleotides. The standard reaction to test for YhdE (d)NTP PPase activity contained the following: 1.5 μM YhdE, 100 or 500 μM substrate, 2 mM Mn^2+^ and 20 mM HEPES buffer pH 7.0 in a 50 μL volume. The samples were incubated at 25°C for 10 minutes. These assays were repeated with the YhdE_E33A mutant. All reactions were analyzed with the P_i_Per Pyrophosphatate Assay Kit. The absorbance at 565 nm was recorded together with a control lacking the enzyme.

### Enzymatic kinetic analysis of YhdE PPase activity

The standard reaction for the kinetic analysis of YhdE dTTP and UTP PPase activity contained the following: 1.5 μM YhdE, 0~300 μM substrate, 2 mM Mn^2+^ and 20 mM HEPES buffer pH 7.0 in a 50 μL volume. The samples were incubated at 25°C for 10 minutes. One unit of enzyme is defined as the amount of the enzyme required for the hydrolysis of 1 μM substrate per minute at 25°C. All reactions including a control of lacking enzyme were analyzed with the P_i_Per Pyrophosphatate Assay Kit. The absorbance at 565 nm was recorded.

### Crystallization

The preliminary crystallization conditions for the YhdE_E33A mutant were screened by the sparse matrix method [19] using standard screening kits. The protein concentration was 10 mg mL^-1^ in Bis-Tris buffer (pH 6.75). The hanging-drop vapor-diffusion method was used. Hanging drops were set up with 2 μL protein solution mixed with 2 μL well solution. The final optimized crystallization condition was 0.1 M magnesium sulfate, 0.1 M MES buffer (pH 6.5), and 10~15% PEG 8000 as the precipitating agent in the reservoir at room temperature. The crystals appeared in six days and grew to full size within two weeks (S1A Fig.). For crystallization of YhdE_E33A in the presence of substrate, dTTP was added to a final concentration of 1 mM in the YhdE_E33A protein solution. The crystals were obtained in the same growth condition, though a different crystal form developed (S1B Fig.).

### Structure determination

The diffraction data sets for the crystal of apo YhdE_E33A and the crystal of YhdE_E33A in the presence of dTTP were collected using in-house facilities, including a Rigaku rotating anode generator and a MarResearch Mar345 imaging plate. All data were processed with HKL2000. Molecular replacement solutions were found by Phaser [20], using the structure of the Maf protein from *B*. *subtilis* as the search model. The remainder of the model was built, and side chains, when appropriate, were corrected manually using Xtalview [21]. All subsequent refinements were carried out with REFMAC5 in the CCP4 suite [22].

### Docking studies

Docking dTTP in YhdE or YhdE_E33A was performed by ADT (http://mgltools.scripps.edu/downloads) and AutoDock (http://autodock.scripps.edu/). The three-dimensional structures of YhdE and dTTP are displayed by using PyMOL (http://www.pymol.org/).

### Strains for cell growth and cell morphology experiments

The *E*. *coli* strain K-12 W3110 (wild-type) and *yhdE*-knockout (KO) strain were kind gifts from the Genome Analysis Project in Japan (http://ecoli.aist-nara.ac.jp), dubbed Keio collection [23].

pGEX-6P-1-*yhdE* and pGEX-6P-1-*yhdE_E33A* were constructed to perform complementation experiment for the KO strain. *yhdE* gene was amplified by polymerase chain reaction using CG**GGATCC**ATGACTTCTCTGTATTTAGCTTCCG as the forward primer and CCG**CTCGAG**TCAGCCGTCATGTTTATCCC as the reverse primer (restriction sites are in bold type). The resulting product was digested with BamHI and XhoI and ligated into the pGEX-6P-1 vector. Both pGEX-6P-1-*yhdE* and pGEX-6P-1-*yhdE_E33A* were expressed respectively in the KO strain by inducing with 0.2 mM isopropyl-β-d-thiogalactoside (IPTG).

### Cell growth conditions

Overnight Luria-Bertani (LB) cultures of strains were diluted with LB media without additional NaCl or glucose, and LB media containing different concentrations of NaCl (250 mM, 500 mM, 750 mM and 1 M) or glucose (5%, 10%, 15%, 20%, w/v) to the same optical density at 600 nm (OD_600_) respectively. The cultures were then incubated with shaking at 37°C for 12 hours. The cell growths were monitored by taking hourly OD_600_ measurements.

### Scanning electron microscopy (SEM)

For scanning electron microscopy sample preparation, strains were cultured overnight at 37°C in LB broth (1 mL). Cells were fixed with 2.5% glutaraldehyde, dehydrated with gradient concentrations of ethanol from 10%~70%, and dropped onto foil that was pasted onto a metal specimen holder after the cells had dried. The SEM images of cells were obtained using a S4800 SEM instrument (Hitachi, Japan).

### Transmission electron microscope (TEM)

For transmission electron microscopy sample preparation, strains were cultured overnight at 37°C in LB broth (1 mL). YhdE was expressed in BL21(DE3) by inducing with 0.2 mM IPTG for 4 hours. Cells were harvested by centrifugation for 10 minutes at 4500×g. After washing three times with PBS (pH 7.4), cells were pre-fixed with 2.5% formaldehyde and 2.5% glutaraldehyde in 0.1 M cacodylate buffer for 1 hour at room temperature (RT). Cells were then washed three times with 0.1 M cacodylate buffer and post-fixed with 1% osmium tetroxide in 0.1 M cacodylate buffer for 100 minutes at RT. After fixation, cells were stained with 1% uranyl acetate for 40 minutes at RT and dehydrated through a series of graded acetone solutions (30%, 50%, 70%, 90%, 10 minutes each, and 100% acetone 3×10 minutes each). After dehydration, cells were infiltrated with a series of embedding resins (Spurr’s resin: 100% acetone, 1:3, 1 hour; Spurr’s resin: 100% acetone, 1:1, 1 hour; Spurr’s resin, 3 hours) and subsequently incubated in an oven at 65°C overnight with Spurr’s resin. Samples were finally sectioned (70 nm slices) and transferred to TEM grids (200 mesh copper grids; EMS Sciences, USA). Before observation, sections were stained with 2% uranyl acetate and lead citrate. Digital images were acquired using a Multiscan CCD camera on a Tecnai spirit microscope at an accelerating voltage of 120 KV.

## Results

### Substrate specificity and enzymatic kinetics of YhdE

**Fig 1:**
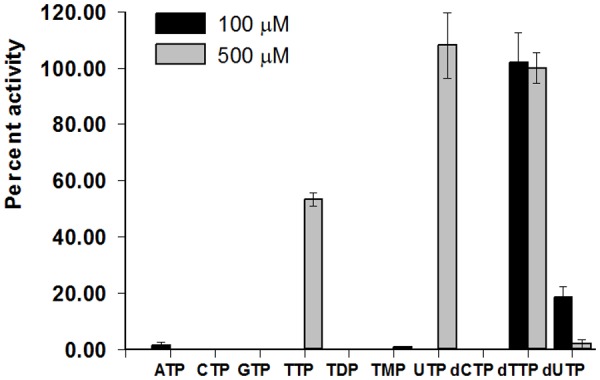
YhdE substrate specificity. Two different substrate concentrations were tested. YhdE (1.5 μM) in the presence of 2 mM Mn^2+^, 20 mM HEPES buffer pH 7.0 and either 100 μM or 500 μM TTP was incubated for 10 minutes at 25°C. The percent activity for each of the different nucleotides tested is shown. Activity is expressed as a percentage of the YhdE activity using dTTP as the substrate at 500 μM. All experiments were completed in triplicate and performed twice. The error bars represent the standard error of the mean.

**Fig 2:**
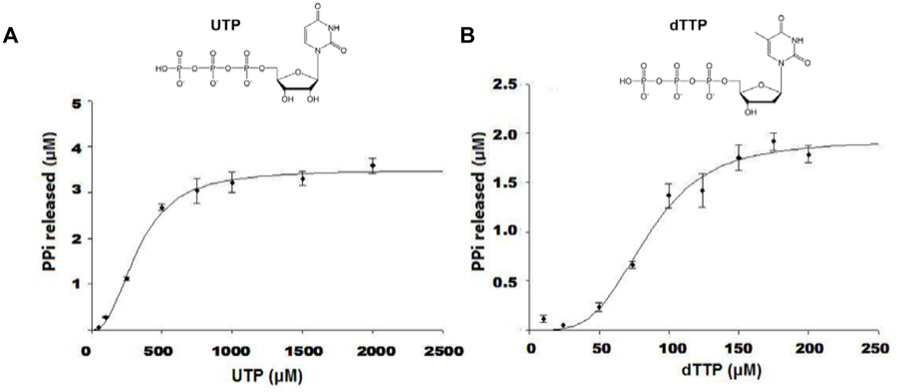
Kinetic analysis of YhdE PPase activity. (A) UTPase activity of YhdE. For kinetic measurements, 1.5 μM YhdE was assayed under standard conditions (2 mM Mn^2+^, 20 mM HEPES buffer pH 7.0, 25°C), along with various concentrations of the substrate UTP. (B) dTTPase activity of YhdE. For kinetic measurements, 1.5 μM YhdE was assayed in standard conditions (2 mM Mn^2+^, 20 mM HEPES buffer pH 7.0, 25°C), along with various concentrations of the substrate dTTP. Kinetic parameters were calculated using SigmaPlot. All experiments were completed in triplicate and performed twice. The error bars represent the standard error of the mean.

To investigate YhdE’s enzymatic activity, recombinant YhdE was expressed and purified to near homogeneity by Ni^2+^ chelation. Subsequent purification by size-exclusion chromatography indicated that YhdE is a dimer in solution with a size of approximately 45 kDa (S2 Fig.). Previous studies of Maf, the *B*. *subtilis* homologue of YhdE, showed that it is has nucleotide-binding capabilities [3], and a more recent study [6] investigated the nucleotide PPase activity of YhdE. Our results showed that YhdE possesses dTTP-specific activity at a substrate concentration of 100 μM. YhdE exhibited significant activity for both UTP and TTP only when substrate concentrations reached 500 μM (Fig. 1). Activity saturation was reached for both UTP and dTTP (Fig. 2, Table 1). The apparent K_m_ values for UTP and dTTP were 0.50 mM and 0.09 mM, respectively. Despite the fact that UTP has a greater apparent k_cat_ (95 s^-1^) than dTTP (43 s^-1^), dTTP still displays greater apparent catalytic efficiency. The apparent k_cat_ /K_m_ values are 191 mM^-1^ s^-1^ and 503 mM^-1^ s^-1^ for UTP and dTTP, respectively. Our analysis also indicated that the apparent Hill’s coefficients for UTP and dTTP are both approximately two.

**Table 1:** Kinetic analysis of YhdE with dTTP and UTP.

Substrate	K_m_ (mM)	V_max_ (mM s^-1^)	k_cat_ (s^-1^)	k_cat_/K_m_ (mM^-1^ s^-1^)	N (Hill)
dTTP	0.09	0.06	43	503	2.2
UTP	0.5	0.14	95	191	1.9

### Conformational flexibility of YhdE and its adaptable active site

To explore the catalytic mechanism of YhdE’s activity and in an attempt to trap substrate to form a complex, we generated the E33A mutant (YhdE_E33A), which abolished YhdE PPase activity [6]. We crystallized YhdE_E33A in the presence and absence of its substrate, dTTP, to determine the conformational changes. Two different crystal forms were obtained; the first one belongs to the *P*4_3_ space group (PDB code 4P0U), while the second one form obtained in the presence of dTTP belongs to the *P*2_1_2_1_2_1_ space group (PDB code 4P0E) (S1 Fig., S1 Table). The overall structure of YhdE_E33A is a dimer and is highly similar to those of members of the Maf protein family (PDB code 2P5X, 4HEB, 2AMH, 4JHC). The subunit structure contains a YhdE_E33A monomer with an α/β fold that has seven α-helices and six extended, mixed β-strands. The six strands form a twisted β-sheet at the center of the protein, connecting two lobes (lobes A and B). A large cavity is located in between the two protein lobes. Most of the conserved residues of YhdE are located inside this cavity, suggesting that it is the active site of this enzyme.

**Fig 3:**
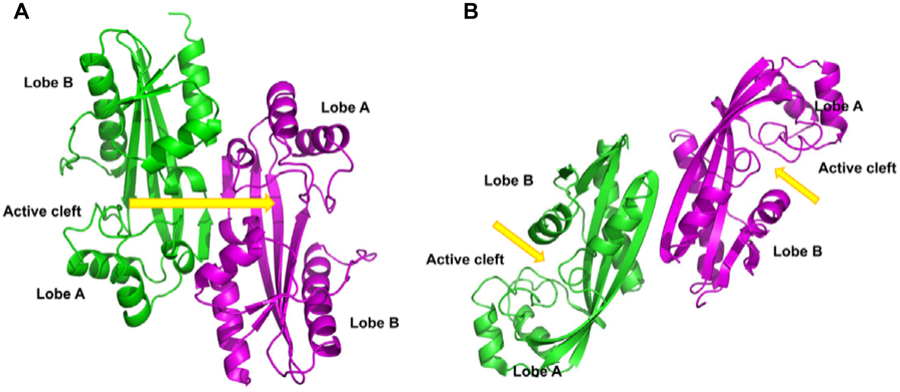
Comparison of YhdE_E33A and Maf dimers. (A) The YhdE_E33A dimer. (B) The Maf dimer. The ribbon diagrams of two monomers are in green and magenta. The active sites are marked by yellow arrows. These figures were made using PyMOL.

Although Maf also forms a dimer in solution, the dimeric mode of YhdE_E33A is different from that of Maf (Fig. 3). In YhdE, the interface occurs at a small β-stand between the two monomers, which places the A lobe of one monomer next to the B lobe of another monomer, bringing the two active sites close together. In *B*. *subtilis*, the Maf interface occurs at the elongated β-stand between the two monomers, which places the B lobe of one monomer next to the B lobe of another monomer, separating their active sites on opposite sides of the Maf dimer. This difference may explain the cooperative nature of the YhdE enzymatic activity; binding of one substrate molecule is likely to affect the binding of another substrate molecule given the proximity of the active sites in the dimer.

**Fig 4:**
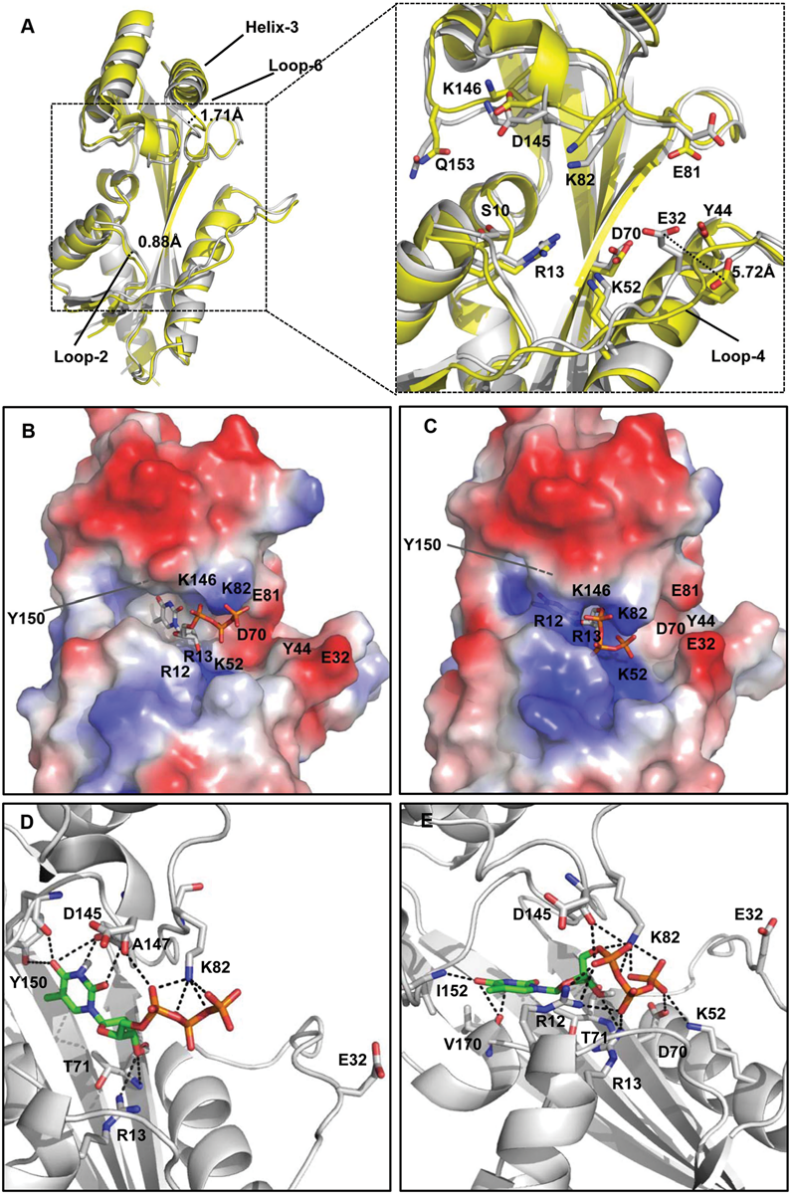
Structure details. (A) The overall aligned structure of YhdE_E33A and detailed structure of substrate-binding pocket in YhdE_E33A open conformation (yellow, PDB code 4P0U) and closed conformation (white, PDB code 4P0E). Residues involved in substrate binding or implicated in the reaction are shown as sticks. Conformational changes are presented based on the distance between the two C-α atoms on the same residue of main chain in loop-6 near helix-3 (1.71 Å) and loop-2 (0.88 Å). The conformational shift of the E32 residue between the open and closed conformations is shown in the detailed figure on the right. Distance measurement was performed by aligning the two conformations and measuring the distance between the two CD atoms in the carboxyl group of the two E32 residues. Comparison of YhdE surface charge in the open (B) and closed conformations (C). Blue and red indicate positive and negative charge distribution on the YhdE surface, respectively. Binding model of dTTP in the YhdE open (D) and closed conformations (E). The amino acids involved in the interaction with dTTP are presented with all carbon atoms colored white. dTTP is presented as all carbon atoms colored green. E32 is also shown in each figure.

Comparison of the structures of YhdE_E33A in the two different space groups revealed substantially different conformations of the cleft formed by the main and side chains of R13, K82, K146, E32, E81 and other related residues located in the active site (Fig. 4A). The structure of YhdE_E33A alone shows an ‘open’ conformation, and the structure of YhdE_E33A crystallized in the presence of dTTP shows a ‘closed’ conformation. The major difference between the two structures is the shape and volume of the cleft pocket and its surface charge distribution. The open conformation is very similar to the structures of Tb-Maf1 (PDB code 2AMH) and YceF (PDB code 4JHC) [6], in which the active sites can accommodate the substrate without steric conflict; E32 points to the exterior of the protein. In contrast, in the closed conformation, YhdE E32 is oriented towards the active site and partially occludes the substrate-binding pocket with its carboxyl group. Thus, E32 can adopt two alternative conformations in YhdE with its carboxyl group either pointing into or out of the substrate-binding pocket, a shift of 5.7 Å (Fig. 4A).

In addition to these conformational shifts, there is also a significant difference with respect to the electrostatic charge in the active site between the two structures (Fig. 4B, 4C). On the right side of the active site, Y44, E32, D70, and E81 form a negatively charged patch. Within the active site, R12, R13, K52, K82 and K146 create a positively charged region, similar to what is observed in Tb-Maf1 and Ycef. However, the charge distributions between the two forms of YhdE are substantially different. The positive charge in the active site below Y150 is stronger in the closed conformation. The negative charge distribution to the right of the positively charged active site is also affected by the conformational differences between the open and closed structures, primarily as a result of the change in the conformation of E32, which points inward in the closed conformation while pointing outward in the open structure.

Crystallization of YhdE in the presence of dTTP allowed us to capture a never-before-observed closed conformation and observe interesting conformational differences relative to the more canonical open structure seen in other family members. However, despite this novel finding, unfortunately, the presence of dTTP was not detected in the closed structure. Thus, we performed a computational study to dock dTTP in both the open and closed structures of YhdE_E33A. The lowest-energy structure was extracted in order to analyze substrate binding in the active site. Interestingly, dTTP could be docked to both conformations but in different orientations (Fig. 4D, 4E). In the open conformation, the orientation of the docked dTTP is consistent with that reported in a previously published work [6], although the thymine base is located in almost the same position in the active site in both the open and closed conformations. Through its base, dTTP interacts with Y150 (both main chain and side chain) and A147 (main chain) in the open form. In contrast, within the closed structure, the methyl group on the thymine of dTTP points to I152 and twists approximately 90°. The positioning of the phosphate groups is quite different as well (Fig. 4D, 4E). Overall, this structure appears to represent a new conformation of YhdE not previously observed. Therefore, we postulate that YhdE has an adaptive active site that can readily accommodate substrates in more than one conformation. The accumulation of positional differences of the active site residues, albeit relatively small (except E32) individually, collectively result in considerable differences in both structure and charge distribution in and around the active site that have implications for substrate recognition and binding.

### YhdE inhibits cell growth in *E*. *coli*

**Fig 5:**
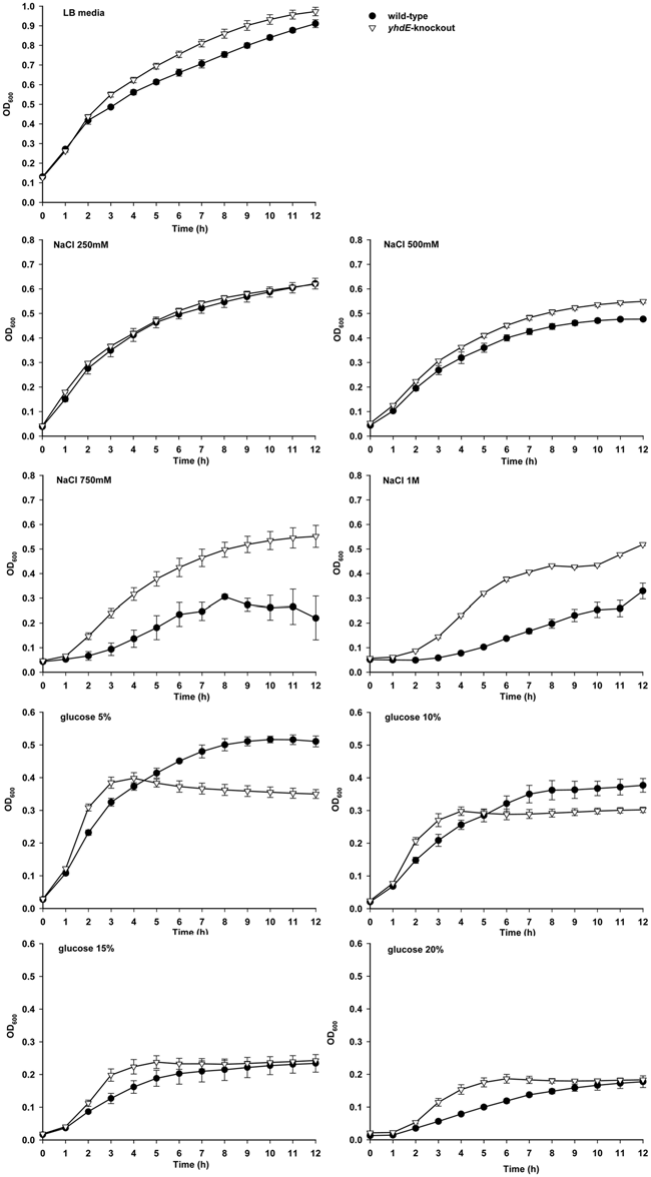
Effect of *yhdE* gene on cell growth. Growth curves for wild-type *E*. *coli* K-12 W3110 (dots) and *yhdE*-knockout (KO) strain (triangles) in basic LB media and different physiological conditions were constructed from OD_600_ measurements under aerobic conditions at 37°C. There was no obvious difference in the growth tendencies of these two strains cultured in LB media or in LB media with 250 mM and 500 mM NaCl. When 750 mM or 1 M NaCl was added to LB media, the wild-type strain significantly increased the cell-doubling time and prolonged the lag phase of the cell-growth curve. In contrast, the KO strain showed faster growth relative to the wild-type strain. When we added 5% (w/v) glucose to LB media, the KO strain grew faster than wild-type prior to the stationary phase and wild-type strains surpassed KO strain subsequently. With increased glucose concentrations, the growth of wild-type cells gradually slowed. All experiments were completed in triplicate and performed twice. The error bars show the standard error of the mean.

To determine the effect of YhdE on cell growth, we systematically investigated growth profiles of *E*. *coli* strains including *E*. *coli* K-12 W3110 (wild-type) and the *yhdE*-knockout (KO) strain under varying physiological conditions (Fig. 5). We found that in basic LB media, the wild-type strain grew slightly slower than the KO strain, and there was no significant difference between the growth tendencies of these two strains. When we added 250 mM or 500 mM NaCl to LB media, the difference in the growth curves of these two strains was similar to that of cultures grown in basic LB media. When the concentration of NaCl increased to 750 mM, the wild-type strain significantly increased cell doubling time and elongated the lag phase of the cell-growth curve, in which phase enlargement, adjustment and synthesis occur. In contrast, the growth of the KO strain was faster than the wild-type strain in this condition. This trend was more conspicuous when the concentration of NaCl was raised to 1 M. When we added 5% (w/v) glucose to LB media, the KO strain grew faster than the wild-type prior to the stationary phase, at which point the wild-type strain surpassed the KO strain. With increased glucose concentrations, the growth of the wild type gradually slowed, characterized by an obvious retard in the lag and exponential-growth phases. The growth rate of the wild-type strain was even less than that of the KO strain during the stationary phase at 15% and 20% glucose. These results indicate that YhdE negatively affects cell growth, especially at high concentrations of NaCl (750 mM and 1 M) and glucose (15% and 20%), during the lag and exponential-growth phase.

**Fig 6:**
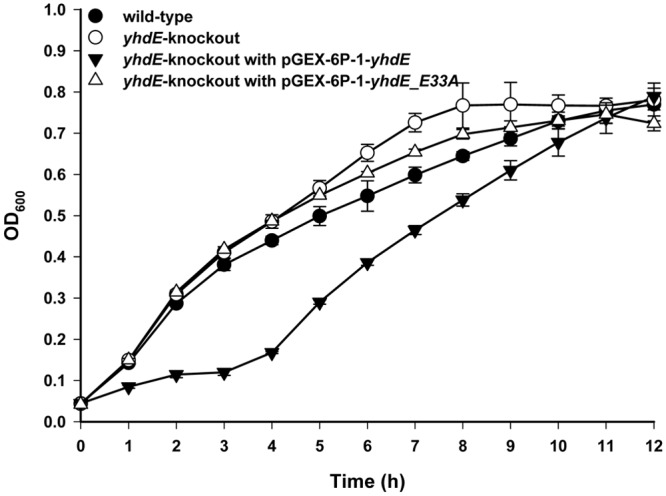
Complementary effect of YhdE on cell growth of *yhdE*-knockout strain. Growth curves for wild-type *E*. *coli* K-12 W3110 (black dots), *yhdE*-knockout strain (empty dots), *yhdE*-knockout strain harboring pGEX-6P-1-*yhdE* (black triangles) or pGEX-6P-1-*yhdE_E33A* (empty triangles) in basic LB media were plotted from OD_600_ measurements under aerobic conditions at 37°C. 0.2 mM IPTG was added to induce the expression of YhdE and YhdE_E33A.

When YhdE was overexpressed in the KO strain, the growth rate was significantly slowed down and the lag phase of cell growth curve was especially retarded. When YhdE_E33A, the single site mutant which have lost PPase activity, was overexpressed in the KO strain, the inhibition function almost disappeared (Fig. 6). This result confirms that YhdE is an inhibitor of cell growth and indicates that the PPase activity of YhdE is associated with the inhibition function of YhdE in cell growth.

### YhdE is essential to maintaining the rod shape of *E*. *coli*

**Fig 7:**
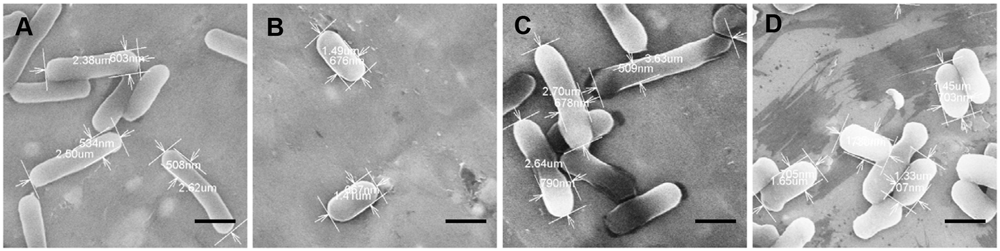
Scanning electron micrographs of *E*. *coli* strains. (A) Wild-type strains; (B) *yhdE*-knockout (KO) strain; (C) KO strain harboring pGEX-6P-1-*yhdE*; (D) KO strain harboring pGEX-6P-1-*yhdE_E33A*. Cells were prepared for scanning electron microscopy as described in the Methods. Compared with the wild-type cells, the KO cells were more spherical, shorter in length. When YhdE expressed in KO strain, the cell length recovered to normal level as wild-type strain. Mutant YhdE_E33A without PPase activity cannot complement the shape and length of cell. Scale bars represent 1 μm.

To examine YhdE’s effect on cell morphology, we performed scanning electron microscopy (SEM) and transmission electron microscopy (TEM) experiments using wild-type, YhdE KO and overexpression strains of *E*. *coli*. As shown in the SEM images, the KO cells exhibited a more spherical shape relative to the rod-shaped wild-type cells (Fig. 7B). Cells dimension measurements indicate that KO cell sizes were shorter in length (1.49 μm *vs* 2.62 μm), yet slightly wider in width (676 nm *vs* 508 nm). Thus, YhdE appears to have a significant effect on cell shape.

When YhdE was overexpressed in the KO strain, the length of cell recovered (2.70 μm) and the rod shape of cell reappeared (Fig. 7C). When complemented with YhdE_E33A, the KO strain did not elongate and still remained shorter morphology (1.45 μm) (Fig. 7D). These results confirm that YhdE is important in maintenance of cell shape and indicate that the PPase activity was essential in this cell function.

**Fig 8:**
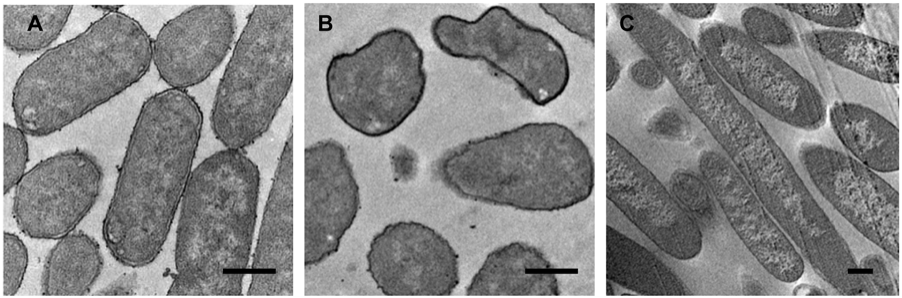
Transmission electron micrographs of *E*. *coli* strains. (A) Wild-type *E*. *coli* strain. (B) *yhdE*-knockout (KO) strain. (C) YhdE overexpression strain. The wild-type cells exhibited a regular rod-like shape with a double-membrane structure and a smooth cell wall. The KO cells displayed an irregular round shape and were less uniform. In contrast, YhdE overexpression cells appeared quite elongated. Scale bars represent 500 nm.

TEM images of ultrathin sections prepared from the cells revealed extensive morphological differences among the wild-type, KO and YhdE overexpression strains. Wild-type cells exhibited a regular rod-like shape with an obvious double-membrane structure and a smooth cell wall. The nucleoid region could even be observed at higher magnification (Fig. 8A). In KO cells, the overall cell size was comparable to wild-type cells, but most of these cells displayed an irregular spherical shape and were less uniform. The plasma membrane was significantly smaller, and the double-membrane structure was completely absent. Due to differential responses to staining, the KO cell walls appeared much darker than those of wild-type cells (Fig. 8B). In contrast, YhdE overexpression cells appeared quite elongated (Fig. 8C), which is consistent with the results of overexpression of Maf in *B*. *subtilis* [1]. These morphological changes suggest that YhdE overexpression somehow blocks cell division. The altered cell membrane and irregular shape of KO cells, just like oncocytes in eukaryotes, possibly resulting from fast and uncontrolled cell division.

## Discussion

### YhdE specificity for dTTP

A recent paper reported the PPase activity of a panel of Maf proteins [6]. In our study, we extend this report and show that dTTP is a slightly better substrate than UTP or TTP for YhdE. The catalytic efficiency, k_cat_/K_m_, is approximately 2.5-fold greater for dTTP than UTP. The Hill’s coefficients are approximately two, indicating that two substrate molecules follow a non-Michaelis-Menten model and participate in positive cooperative binding to YhdE. The fact that YhdE exists as a dimer in solution suggests that each YhdE dimer can bind up to two substrates at any one time. To investigate the observed mechanism of substrate recognition, specificity and cooperatively, we solved the structures of the inactive mutant YhdE_E33A. The structure shows that, in the YhdE dimer, the two active sites are positioned close to each other, therefore making cooperativity possible.

### Substrate reorganization and catalytic mechanism of YhdE

As previously suggested, the methylated substrate (dTTP) introduces a certain degree of hydrophobic interactions with the neutral surface at the edge of the binding pocket. Based on the open-conformation docking model, the base group is prone to interacting with this neutral surface in order to stabilize dTTP during the rearrangement of the active site after substrate binding. Other similar substrates, such as dUTP, which lack this methyl group, may slip into alternative unbound conformations.

After substrate binding mediated by hydrogen-bond interactions in the active site, the reaction would proceed with a conformational change involving the E32, and possibly E33, carboxyl groups which point out of the active site, resulting to an increase in the free energy of the entire system. Due to the repulsive interactions of the E32 carboxyl group and the substrate, the electron density between the α-β phosphates is greater than that between β-γ phosphates, leading to the cleavage of the α-β bond instead of the β-γ bond and the subsequent dissociation of the substrate. The resultant product exhibits a different charge distribution relative to the original reactant. Consequently, the repulsive force of the reactive center becomes greater than the hydrogen-bond interactions involved in binding, resulting in the disassociation of the reaction product from the active site.

### YhdE acts as a cell-division inhibitor in *E*. *coli*

When organisms face various growth-limiting stress conditions, they often turn on self-regulation systems to ensure survival and foster the evolutionary process. Several proteins have been identified as cell-division inhibitors and play an important role in this self-regulation system, such as SulA [24], DicB [25] and YneA [26] in bacteria. Previous studies show that Maf is also implicated in the inhibition of cell division. In this study, we focused our efforts on YhdE, a Maf-like protein in *E*. *coli* for which a cellular function has yet to be determined. First, we demonstrated that YhdE is a cell-growth inhibitor based on cell-growth behavior. Inhibition of cell growth by YhdE increased under conditions of stress, such as high salt and high sugar and primarily occurred during the lag and exponential growth phases. These results collectively indicate that YhdE may play a role as a regulator under stress conditions. Second, the filamentous phenotype exhibited by the YhdE overexpression strain confirms the role of YhdE in cell division arrest. In contrast, *yhdE*-knockout cells exhibited a collapse in the rod shape as well as membrane spoiling, indicating that YhdE is essential to the maintenance of cell shape. The aberrant cell morphologies observed in *yhdE*-knockout cells indicate that the cells are unable to self-regulate as a result of its loss of the ability to sense a lack of nutrients or irreparable damage, leading to premature cell division, immoderate and aberrant cell growth.

### PPase activity and cell growth inhibitor

The fact that inactive YhdE mutant lacking PPase activity could not regulate cell growth implies that there is a relationship between YhdE inhibitor function in cell growth and PPase activity. Although the roles of YhdE in inhibiting DNA and RNA synthesis, as well as its housekeeping function, have been suggested because dTTP is an important nucleotide in DNA replication, it is noteworthy that dTTP and UTP are also important precursors in cell wall synthesis [27, 28]. Thus, the fact that both dTTP and UTP are excellent substrates of YhdE suggests that YhdE may negatively regulate the rate of cell wall synthesis through hydrolysis of required precursors, which may serve as another means to regulate the cell-cycle checkpoints.

## Supporting Information

S1 Fig Crystals and diffraction image. (A) YhdE_E33A crystal (*P4*_*3*_). (B) Crystal of YhdE_E33A in the presence of dTTP (*P2*_*1*_ *2*_*1*_ *2*_*1*_).
(TIF)

S2 Fig Purification of YhdE. (A) Fast Protein Liquid Chromatography Purification. Absorbance at 280 nm (y-axis) as a function of the volume eluted (x-axis). The buffer used was Bis-Tris buffer, pH 6.75. YhdE eluted at approximately 81 mL, corresponding to a molecular weight of approximately 45 kDa, which corresponds to a YhdE dimer. (B) SDS-PAGE of purified YhdE. Purified YhdE protein sample run on an SDS-PAGE after concentration to approximately 20 mg mL^-1^. Lane 1: molecular weight ladder; Lane 2: YhdE protein sample. YhdE has a molecular weight of approximately 22 kDa, equivalent to that of the monomeric YhdE.
(TIF)

S1 Table Statistics of X-ray diffraction data. (XLSX)
