# Supplementary figures and images for: Insights into the Cellular Function of YhdE, a Nucleotide Pyrophosphatase from Escherichia coli
Source: PLoS One. 2015 Feb 6;10(2):e0117823. doi: 10.1371/journal.pone.0117823 (PMC4319933; doi:10.1371/journal.pone.0117823)

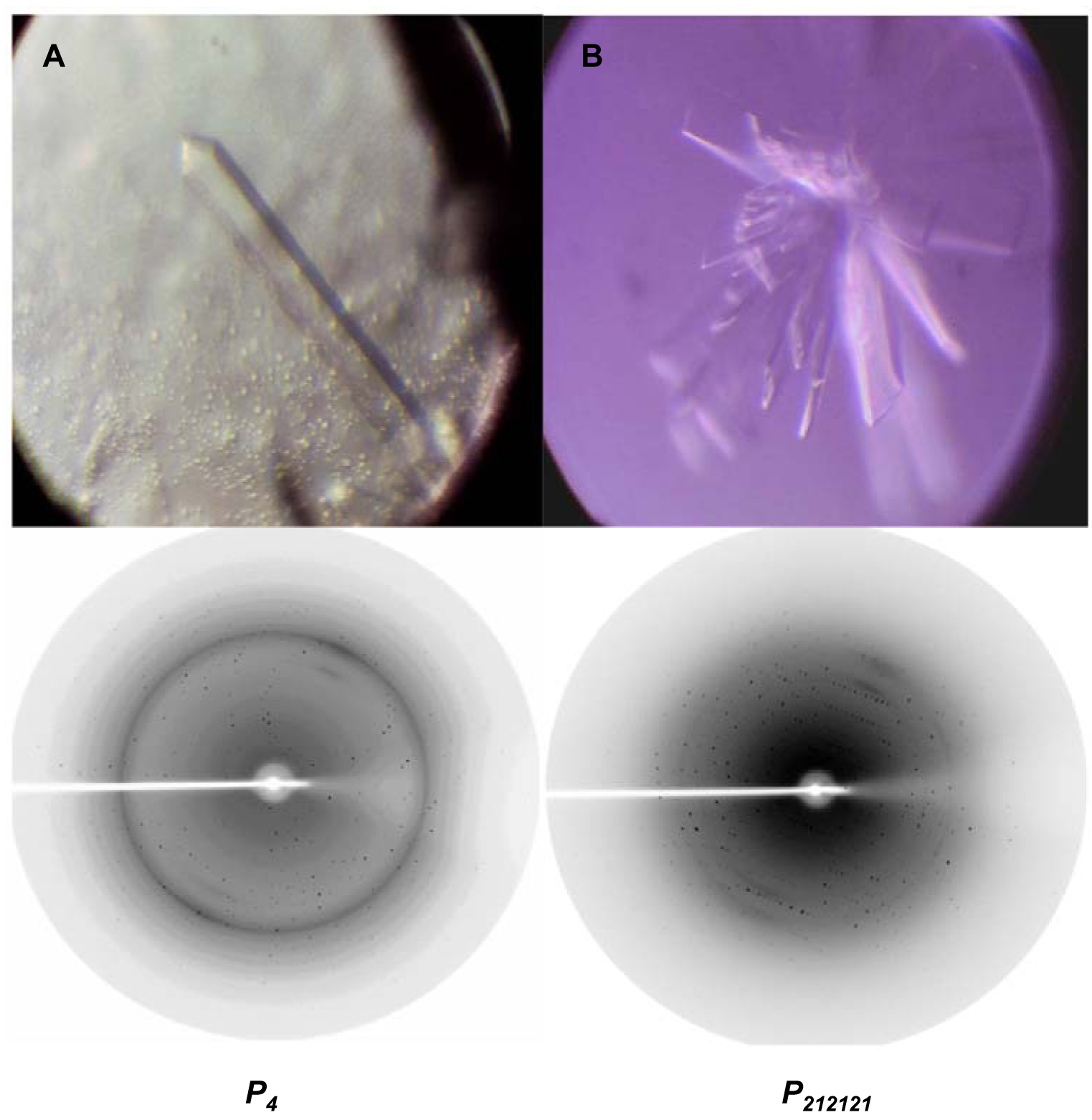

Supplement: S1 Fig — (A) YhdE_E33A crystal (P4 3). (B) Crystal of YhdE_E33A in the presence of dTTP (P2 1 2 1 2 1). (TIF) [file pone.0117823.s001.tif]

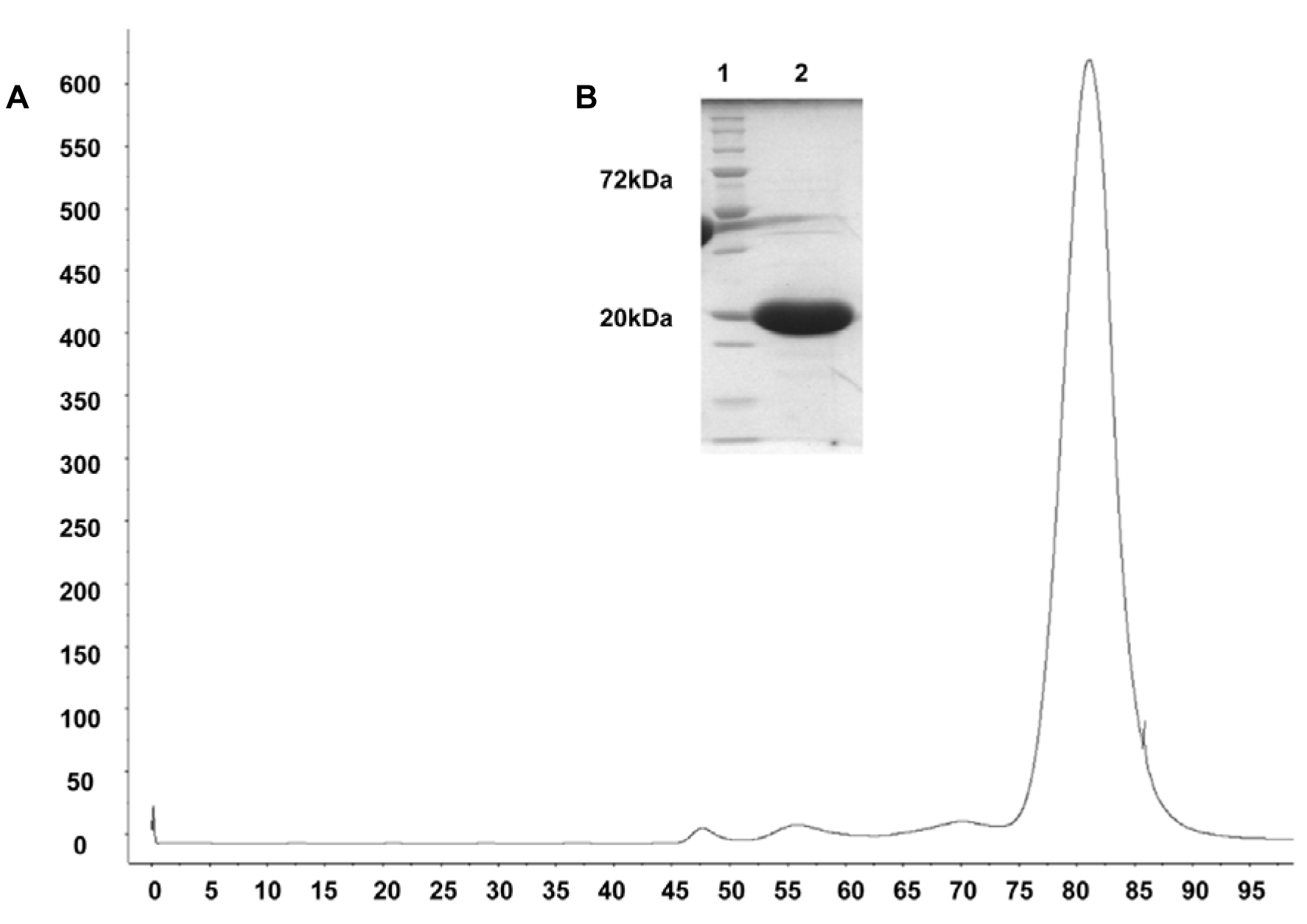

Supplement: S2 Fig — (A) Fast Protein Liquid Chromatography Purification. Absorbance at 280 nm (y-axis) as a function of the volume eluted (x-axis). The buffer used was Bis-Tris buffer, pH 6.75. YhdE eluted at approximately 81 mL, corresponding to a molecular weight of approximately 45 kDa, which corresponds to a YhdE dimer. (B) SDS-PAGE of purified YhdE. Purified YhdE protein sample run on an SDS-PAGE after concentration to approximately 20 mg mL-1. Lane 1: molecular weight ladder; Lane 2: YhdE protein sample. YhdE has a molecular weight of approximately 22 kDa, equivalent to that of the monomeric YhdE. (TIF) [file pone.0117823.s002.tif]
